# Neuronal Ceroid Lipofuscinoses: Connecting Calcium Signalling through Calmodulin

**DOI:** 10.3390/cells7110188

**Published:** 2018-10-29

**Authors:** Sabateeshan Mathavarajah, Danton H. O’Day, Robert J. Huber

**Affiliations:** 1 Department of Pathology, Dalhousie University, Halifax, NS B3H 4R2, Canada; smathavarajah@trentu.ca; 2 Department of Biology, University of Toronto Mississauga, Mississauga, ON L5L 1C6, Canada; danton.oday@utoronto.ca; 3 Department of Cell and Systems Biology, University of Toronto, Toronto, ON M5S 3G5, Canada; 4 Department of Biology, Trent University, Peterborough, ON K9L 0G2, Canada

**Keywords:** batten disease, neuronal ceroid lipofuscinosis, calmodulin, calmodulin-binding proteins, calmodulin-binding domains, calcium

## Abstract

Despite the increased focus on the role of calcium in the neuronal ceroid lipofuscinoses (NCLs, also known as Batten disease), links between calcium signalling and the proteins associated with the disease remain to be identified. A central protein in calcium signalling is calmodulin (CaM), which regulates many of the same cellular processes affected in the NCLs. In this study, we show that 11 of the 13 NCL proteins contain putative CaM-binding domains (CaMBDs). Many of the missense mutations documented from NCL patients overlap with the predicted CaMBDs and are often key residues of those domains. The two NCL proteins lacking such domains, CLN7 and CLN11, share a commonality in undergoing proteolytic processing by cathepsin L, which contains a putative CaMBD. Since CaM appears to have both direct and indirect roles in the NCLs, targeting it may be a valid therapeutic approach for treating the disease.

## 1. Introduction

The first clinical diagnosis of Batten disease was in 1903, and since then, great strides have been made in our understanding of the cellular and molecular mechanisms underlying this devastating neurological disorder [1]. Batten disease, which is clinically known as neuronal ceroid lipofuscinosis (NCL), is a form of neurodegeneration that has a global distribution and affects people of all ages [2]. Clinical manifestations of the disease include vision loss, seizures, progressive loss of motor function and cognitive ability, and a reduced lifespan [3]. The only clinically-approved therapeutic for the NCLs is Brineura, which is an enzyme replacement therapy specific for only one subtype of the disease (i.e., CLN2 disease) [4]. The absence of therapeutics stems from our poor understanding of NCL proteins and their primary functions. In total, 13 genetically distinct genes are linked to the disease (*CLN1–8*, *CLN10–14*) [2]. These 13 genes encode enzymes (CLN1, CLN2, CLN5, CLN10, and CLN13), transmembrane proteins (CLN3, CLN7, and CLN12), membrane proteins that localize to the endoplasmic reticulum (CLN6 and CLN8), cytoplasmic proteins (CLN11 and CLN14), and a protein found on synaptic vesicles (CLN4) [2]. Along with distinct localizations, the NCL proteins have been linked to fundamental cellular processes, including sphingolipid metabolism, protein degradation, and lysosomal pH homeostasis, among others [5,6,7,8,9,10]. Since mutations in NCL proteins cause nearly identical clinical phenotypes, they are thought to participate in shared or convergent biological pathways [11]. However, the common link between the proteins has yet to be revealed.

For clues to explain the molecular networking of NCL proteins, research groups have looked for reoccurring phenotypes in NCL patients [12]. The accumulation of mitochondrial ATP synthase subunit c is one such cellular pathology observed in all the NCLs; however, the cause of the protein accumulation is unknown [13]. Coinciding with this protein accumulation is the degeneration of excitable cells, such as neurons and photoreceptor cells [13]. In a previous study, it was proposed that subunit c accumulation caused altered calcium signalling in electrically excitable neurons and photoreceptor cells [13]. Recent evidence continues to build a framework around aberrant calcium signalling being linked to NCL pathology [14,15,16,17,18]. For example, it has been proposed that amlodipine, a drug which reduces intracellular calcium, could function as a therapeutic for NCL patients. This is based on observations that amlodipine reduced the abnormally high levels of apoptosis in rat neuronal cells where *CLN3* expression was knocked down [19]. Consistent with these findings, phenotypes observed in *Dictyostelium* and *C. elegans* models of CLN3 disease can be suppressed by reducing the levels of intracellular calcium [20,21,22]. These findings highlight a conserved relationship between calcium signalling and the NCL proteins from evolutionarily distant eukaryotes to humans.

Eukaryotic cells pump calcium into and out of their organelles to mediate a variety of cellular processes, including nerve cell transmission (i.e., generating action potentials), motility, exocytosis, apoptosis, and transcription [23]. A key protein that links calcium signalling to these processes is calmodulin (CaM), the primary sensor of calcium in the cell [24]. In the presence of calcium, CaM undergoes a conformational change allowing it to bind and regulate a variety of CaM-binding proteins (CaMBPs) [24]. Proteomic screens of the mammalian brain have identified many calcium-dependent CaMBPs with diverse functions [25,26]. Among these CaMBPs are neuronal nitric oxide synthase, calcineurin A, calcium/CaM-dependent protein kinase II (CaMKII), calcium/CaM-dependent protein kinase kinase, and various calcium channel proteins. Since CaM acts as a critical link between these proteins, it raises the possibility that it may also perform a similar function for the pathways regulating NCL protein function.

## 2. NCL Proteins Influence Cellular Pathways That Are Regulated by CaM

**Figure 1:**
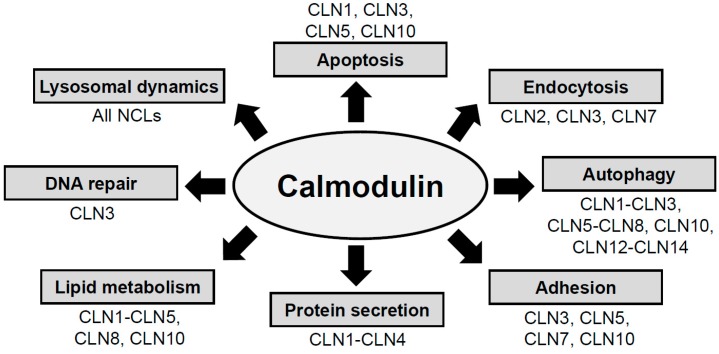
Calmodulin regulates a variety of cellular processes. In two articles—an early one by Chafouleas [27] and a recent one by Berchtold and Villalobo [28]—the diversity of calmodulin-regulated processes (boxed areas: lysosomal dynamics, apoptosis, endocytosis, autophagy, adhesion, protein secretion, lipid metabolism, and DNA repair, among others) were reviewed. For each of these processes, the NCL proteins that are linked to them are noted adjacent to the boxes (e.g., Apoptosis, CLN1, CLN3, CLN5, and CLN10).

CaM regulates molecular pathways linked to autophagy, apoptosis, adhesion, endocytosis, protein secretion, lipid metabolism, lysosomal dynamics, and DNA repair (Figure 1) [24,27,28]. Intriguingly, these cellular processes are also affected in various subtypes of NCL (Figure 1) [19,21,29,30,31]. As further support for the involvement of CaM in the NCLs, Purkinje cell protein 4 (PCP4, also known as PEP19), which modulates the activity of CaMKII, was found to be the most downregulated transcript in a mouse model of CLN1 disease [32]. In addition, the expression of CaMKII was increased in a mouse model of CLN5 disease [33]. Finally, another CaMBP, CaM-dependent protein kinase type 1D, was present in decreased amounts in brain samples from CLN4 disease patients [10]. Interestingly, CaMKII and other CaMBPs play a central role in the progression of Alzheimer’s disease and recent work has linked Alzheimer’s to mutations in CLN5 [34,35]. However, how these CaMBPs affect NCL related pathways has yet to be studied.

## 3. NCL Proteins Have Putative Binding Domains for CaM

CaM binds to CaMBPs via CaM-binding domains (CaMBDs) present in target proteins [25]. CaMBDs have various motifs that enable interactions with CaM; these motifs can be calcium-dependent, which require calcium ions for the interaction, or calcium-independent (Table 1) [36,37]. Calcium-dependent CaM-binding motifs depend on the positioning of hydrophobic residues in the amino acid sequence of target proteins (e.g., the 1–5–10 motif, [FILVW]xxx[FAILVW]xxxx[FILVW]) (Table 1). Calcium-independent CaM-binding motifs are characterized by IQ or IQ-like motifs (I, isoleucine; Q, glutamine) (Table 1) [36,37]. Non-canonical CaMBDs have also been identified, but these are too numerous to list in this article [37].

Our theoretical analysis of potential CaMBDs in the NCL proteins was based upon a previous study that identified putative CaMBDs in the major proteins associated with Alzheimer’s disease [36]. Subsequently, those researchers, and others, experimentally verified the CaM-binding of many of the identified proteins, thereby validating the theoretical approach that was applied [34,38]. In this study, we used the CaM Target Database (http://calcium.uhnres.utoronto.ca/ctdb/ctdb/home.html) to determine if the 13 NCL proteins contain putative CaMBDs [26]. The CaM Target Database scans for the presence of canonical CaM-binding motifs within the amino acid sequence of a suspected CaMBP and is over 90% accurate in predicting a true CaMBD [26]. In our analysis, we disregarded any predicted CaMBDs that fell within transmembrane domains, a common source of error in identifying CaMBDs. Our analysis revealed that 11 of the 13 NCL proteins have putative CaMBDs that belong to different motif subtypes (Table 2).

All 11 NCL proteins that contain putative CaMBDs have calcium-dependent binding motifs (Table 2). The only protein that also has the potential to bind CaM through a calcium-independent mechanism (via an IQ-like motif) is CLN14 (Table 2). Calcium-dependent motifs include the 1–10, 1–5–10, 1–14, 1–8–14, and 1–16 motifs, which are based on the positioning of hydrophobic residues (discussed above in Table 1 and Table 2). In the entire set of identified CaMBDs, the 1–10 (24%; 10/42), 1–14 (21%; 9/42), and 1–16 (29%; 12/42) motifs comprise the majority of identified CaMBD motifs (Table 2). Based on these observations, it appears that calcium signalling may be the primary mechanism governing the regulation of NCL proteins by CaM. Another important revelation is the presence of multiple binding motifs within the CaMBD regions of 9 of the NCL proteins (CLN5 and CLN8 each have a single CaMBD motif, Table 2). Interestingly, many of the predicted CaMBD regions for NCL proteins have multiple motifs of the same type. This is true for CLN2 and CLN13, which each contain multiple 1–16 motifs (Table 2). Since CaM has been shown to bind to peptides with multiple CaM-binding motifs, these overlapping canonical motifs increase the likelihood of an interaction with CaM [37]. In total, these findings suggest that the NCL proteins are likely targets for CaM binding.

In our analysis, we found that CLN7 and CLN11 lack putative CaMBDs (Table 2). Since all the other NCL proteins have potential CaMBDs, we considered other mechanisms that might link CLN7 and CLN11 to CaM. An aspect shared between the two proteins is that they are both proteolytically processed [39,40]. Recent work has shown that proteolytic processing of progranulin (PGRN) into granulin (CLN11) is performed by cathepsin L (CTSL) [41]. Similarly, CLN7 is also processed by CTSL [40]. Cathepsins have been established as key functionaries in the pathology underlying NCL, since mutations in cathepsin D (*CTSD*) and cathepsin F (*CTSF*) cause specific subtypes of the disease (CLN10 and CLN13 disease, respectively) [2]. Interestingly, expression of CTSL reverses the degeneration of neurons in cathepsin B/L double-knockout mice [42]. Furthermore, a relationship between CaM and CTSL has been discussed in previous work [43]. More specifically, the expression of CTSL was shown to be regulated by the CaMBP calcineurin in murine C2C12 skeletal myoblasts [43]. Based on the above findings, we used the CaM Target Database to assess the presence of putative CaMBDs in CTSL. Our analysis revealed a putative calcium-dependent CaMBD in CTSL with two overlapping motifs (1–10 and 1–14 motifs, Table 2). Based on these findings, it is possible that CLN7 and CLN11 are indirectly regulated by CaM through its interaction with CTSL.

**Figure 2:**
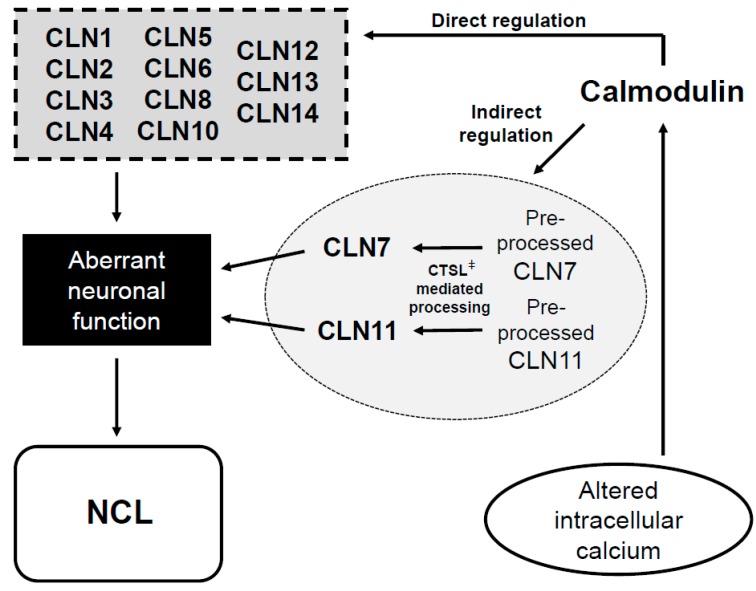
Proposed model linking calmodulin to neuronal ceroid lipofuscinosis. Altered levels of intracellular calcium underlie neuronal ceroid lipofuscinosis (NCL). Since calmodulin (CaM) is the primary downstream target of calcium, in turn, its binding proteins (i.e., CaMBPs) will be involved in facets of the disease. Eleven proteins linked to NCL contain putative CaM-binding domains (CaMBDs) (CLN1–6, CLN8, CLN10, and CLN12–14), suggesting a direct interaction with CaM. Cathepsin L (CTSL), an enzyme that processes CLN7 and CLN11, also contains a CaMBD. Thus, CaM is capable of directly regulating the NCL proteins or indirectly regulating them through CTSL. Mutations in NCL proteins affect neuronal function and results in neurodegeneration. Arrows indicate the links between calcium, CaM, the NCL proteins, and NCL. ^ǂ^ Non-NCL protein.

Direct interactions with CaM are possible for 11 of the 13 NCL proteins (Figure 2), whereas it would likely occur through an indirect interaction via CTSL (Figure 2) if CaM were to regulate CLN7 and CLN11. Our findings indicate that the NCL proteins converge with CaM, which may serve to regulate their functions (Figure 2). In NCL patients, the altered levels of intracellular calcium could influence processes regulated by CaM. Therefore, CaM may play an important role in not only linking the NCL proteins to each other, but also in the pathogenesis of NCL.

## 4. Clinical Relevance for the Presence of CaMBDs in NCL Proteins

Having identified CaMBDs in 11 of the 13 NCL proteins, it was critical to assess the relation of these domains to the disease. Thus, we referenced the list of missense mutations documented from NCL patients for each subtype of the disease (NCL Mutation and Patient Database, https://www.ucl.ac.uk/ncl/mutation.shtml) and analyzed whether these mutations fell within the putative CaMBDs we identified [44]. We focused our analysis on missense mutations resulting from point mutations, since this would identify amino acids that are critical for the normal function of each NCL protein. Our analysis revealed that many deleterious missense mutations map to residues located within the predicted CaMBDs for CLN1, CLN2, CLN3, CLN5, CLN6, and CLN8 (Table 3). In some cases, NCL patient mutations mapped directly to the key residues involved in forming the CaMBD that facilitates the interaction with CaM (Table 3). One thing to note is that for CLN3 disease patient mutations, 21% (4/19) of the documented mutations map to the putative CaMBD were identified. For CLN1 patient mutations, 17% (5/29) of the documented mutations mapped to the putative CaMBDs. In total, our findings suggest that the CaMBDs may be critical for NCL protein function.

The revelation that patient mutations overlap with the putative CaMBDs in the NCL proteins suggests that CaM may play a significant role in the pathology underlying NCL. Targeting CaM and its CaMBPs has shown some success as a potential treatment of Huntington’s disease. In Huntington’s disease, post-translational modifications of huntingtin are carried out by transglutaminase and these modifications contribute to the aggregation of the huntingtin protein. Intriguingly, huntingtin is a CaMBP, and its binding to CaM can be disrupted using a synthetic peptide equivalent in sequence to the region of CaM spanning residues 76–121 (i.e., competitive inhibition) [45]. More specifically, the synthetic peptide was expressed in differentiated neuroblastoma cells (SH-SY5Y) cells and a Huntington’s disease mouse model [45,46]. In both cases, this led to reduced levels of transglutaminase-modified huntingtin and cytotoxicity, thereby leading to neuroprotection. A similar approach of targeting CaM in established models of NCL could yield similar results opening the possibility that CaM and/or CaM-regulated signalling may serve as a therapeutic target for the NCLs [47]. Additionally, it will be key for future research to examine and validate the binding of CaM to the NCL proteins, as well as assess the effects of mutations in NCL proteins on the binding to CaM. Most importantly, if CaM acts to regulate NCL protein function, this could explain how the NCL proteins link to one another [11]. Therefore, further examination of this relationship will enhance our understanding of the mechanisms regulating NCL protein function, which may fuel the development of novel therapies to treat the disease.

## 5. Conclusions

It is currently not known how the 13 NCL proteins are connected at the molecular level. Our analysis revealed that 11 of the 13 NCL proteins have putative calcium-dependent CaMBDs that may facilitate interactions with CaM. The two NCL proteins that lack putative CaMBDs, CLN7 and CLN11, are processed by CTSL, which contains a putative CaMBD. Taken together, these findings suggest that the NCL proteins are linked to CaM, a key regulator of calcium signalling. By identifying putative CaMBDs in the NCL proteins, we determined that a number of patient mutations map to these domains, which opens the door to examine the potential role of CaM in NCL pathogenesis.

## Figures and Tables

**Table 1:** Canonical calmodulin-binding motifs.

**Calcium-dependent calmodulin-binding motifs**	
***1–10 Subclasses***	
1–10	(FILVW)xxxxxxxx(FILVW)
1–5–10	(FILVW)xxx(FAILVW)xxxx(FILVW)
Basic 1–5–10	(RK)(RK)(RK)(FAILVW)xxx(FILV)xxxx(FILVW)
***1–14 Subclasses***	
1–14	(FILVW)xxxxxxxxxxxx(FILVW)
1–8–14	(FILVW)xxxxxx(FAILVW)xxxxx(FILVW)
Basic 1–8–14	(RK)(RK)(RK)(FILVW)xxxxxx(FAILVW)xxxxx(FILVW)
1–5–8–14	(FILVW)xxx(FAILVW)xx(FAILVW)xxxxx(FILVW)
***1–16 Subclasses***	
1–16	(FILVW)xxxxxxxxxxxxxx(FILVW)
**Calcium-independent calmodulin-binding motifs**	
***IQ Subclasses***	
IQ	(FILVW)Qxxx(RK)Gxxx(RK)xx(FILVWY)
IQ-like	(FILVW)Qxxx(RK)xxxxxxxx

**Table 2:** List of putative calmodulin-binding domains in proteins linked to neuronal ceroid lipofuscinosis. The Calmodulin Target Database (http://calcium.uhnres.utoronto.ca/ctdb/ctdb/home.html) was used to reveal putative calmodulin-binding domains (CaMBDs) in proteins linked to neuronal ceroid lipofuscinosis (NCL). Numbers indicate the amino acid positions where the domain is predicted to be present for each protein. Motifs were identified based on known canonical CaM-binding motifs, which are listed in Table 1. Bolded residues mark the amino acids, which are required for the identified motif. Underlined residues represent the residues where NCL patient mutations are also present in the CaMBD. CLN7 and CLN11 are excluded, since they lack CaMBDs; however, the CaMBD within cathepsin L (CTSL) is shown.

NCL Protein	Putative CaMBD	Motif
CLN1 Region 1 (159–183)	ICD**F**IRKTLNAGAYSK**V**VQERLVQA	1–14
	ICDF**I**RKTLNAGAYSKV**V**QERLVQA	1–14
	ICDFIRKT**L**NAGAYSKVVQER**L**VQA	1–14
CLN1 Region 2 (207–232)	QERG**I**NESYKKNLMALKKF**V**MVKFLN	1–16
	QERGINESYKKN**L**MALKKF**V**MVKFLN	1–10
CLN2 (332–358)	LSSAY**I**QRVNTELMKAAARG**L**TLLFAS	1–16
	LSSAYIQR**V**NTELMKAAARGLTL**L**FAS	1–16
CLN3 (318–352)	YR**W**YQM**L**YQAG**V**FASRSSLRCCRIRFTWALALLQ	1–5–10
	YRWYQMLYQAGV**F**ASRSSLRCCRIRFT**W**ALALLQ	1–16
	YRWYQMLYQAGV**F**ASRSSLRCCRIR**F**TWALALLQ	1–14
	YRWYQMLYQAGVFASRSS**L**RCCRIRFT**W**ALALLQ	1–10
	YRWYQMLYQAGVFASRSS**L**RCCRIR**F**TWALA**L**LQ	1–8–14
	YRWYQMLYQAGVFASRSSLRCCR**I**RFT**W**ALAL**L**Q	1–5–10
CLN4 (65–87)	A**I**LTD**A**TKRN**I**YDKYGSLGLYVA	1–5–10
	AI**L**TDATKRNIYDKYGS**L**GLYVA	1–16
	AILTDATKRN**I**YDKYGSLG**L**YVA	1–10
CLN5 (57–84)	QGAEMRRGAGAARGRAS**W**CWA**L**ALLW**L**	1–5–10
CLN6 (245–259)	**V**LHQKRKRL**F**LDSNG	1–10
	V**L**HQKRKRLF**L**DSNG	1–10
CLN8 (45–72)	LSSS**L**NATYRSLVAREKVF**W**DLAATRA	1–16
CLN10 Region 1 (166–189)	ASA**L**GGVKVERQ**V**FGEATKQPGIT	1–10
	ASALGGVKVERQV**F**GEATKQPG**I**T	1–10
CLN10 Region 2 (250–275)	DSKYYKGS**L**SYLNVTRKAYWQ**V**HLD	1–14
	DSKYYKGS**L**SYLNVTRKAYWQVH**L**D	1–16
CLN12 (152–179)	EEA**V**SVGQKR**V**LRYYL**F**QGQRYIWIETQ	1–8–14
	EEAVSVGQKR**V**LRYYLFQGQRYI**W**IETQ	1–14
	EEAVSVGQKRV**L**RYYLFQGQRYIW**I**ETQ	1–14
	EEAVSVGQKRVLRYY**L**FQGQRYIW**I**ETQ	1–10
CLN13 (39–80)	LLAPTRFA**L**EMFNRGRAAGTRAV**L**GLVRGRVRRAGQGSLYSL	1–16
	LLAPTRFALEM**F**NRGRAAGTRAVLGL**V**RGRVRRAGQGSLYSL	1–16
	LLAPTRFALEMFNRGRAAGTRAV**L**GLVRGRVRRAGQGS**L**YSL	1–16
	LLAPTRFALEMFNRGRAAGTRAVLGL**V**RGRVRRAGQGSLYS**L**	1–16
CLN14 (148–166)	HLERIVEIARLRA**VQ**RKA**R**FAKLKVCVFKEEMP	IQ-Like
	H**L**ERI**V**EIAR**L**RAVQRKARFAKLKVCVFKEEMP	1–5–10
	HLER**I**VEI**A**RLRA**V**QRKARFAKLKVCVFKEEMP	1–5–10
	HLER**I**VEIARLRAVQRKAR**F**AKLKVCVFKEEMP	1–16
	HLERIVE**I**ARLRAVQRKARFAK**L**KVCVFKEEMP	1–16
	HLERIVEIAR**L**RAVQRKAR**F**AKLKVCVFKEEMP	1–10
	HLERIVEIARLRA**V**QRK**A**RFAK**L**KVCVFKEEMP	1–5–10
	HLERIVEIARLRA**V**QRKARF**A**KLKVC**V**FKEEMP	1–8–14
CTSL (226–247)	VDIPKQEKALMKAVATVGPISV	1–10
	**V**DIPKQEKALMKA**V**ATVGPISV	1–14

**Table 3:** List of patient mutations present within the putative calmodulin-binding domains (CaMBDs) of proteins linked to neuronal ceroid lipofuscinosis (NCL).

NCL Type	NCL Protein	CaMBD (aa)	NCL Patient Mutations Present within CaMBD	Mutations Last Updated
Infantile	CLN1	159–183	p.Gln177Glu, p.Val181Met, p.Val181Leu	28 November 2017
Infantile	CLN1	207–232	p.Leu222Pro, p.Val228Gly	28 November 2017
Late Infantile	CLN2	332–358	p.Glu343Lys, p.Arg339Trp, p.Leu355Pro, p.Thr353Pro, p.Arg339Gln, p.Arg350Trp	13 November 2017
Juvenile	CLN3	318–352	p.Arg334Cys, p.Val330Phe, p.Arg334Trp, p.Val330Ile	28 November 2017
Adult	CLN4	65–87	None documented	26 February 2018
Variant Late Infantile	CLN5	57–84	p.Trp75Arg	26 February 2018
Variant Late Infantile	CLN6	245–260	p.Arg252His, p.Gly259Val, p.Gly259Ser, p.Asp256Glu	26 February 2018
Variant Late Infantile	CLN7	N/A	-	26 February 2018
Variant Late Infantile	CLN8	45–72	p.Arg70His	26 February 2018
Congenital	CLN10	166–189	None documented	26 February 2018
Congenital	CLN10	250–275	None documented	26 February 2018
Adult	CLN11	N/A	-	26 February 2018
Juvenile	CLN12	152–179	None documented	26 February 2018
Adult	CLN13	39–80	None documented	4 December 2017
Infantile	CLN14	148–166	None documented	4 December 2017

## Abbreviations

CaM	Calmodulin
CaMKII	Calcium/CaM-dependent protein kinase II
CaMBDs	CaM-binding domains
CaMBPs	CaM-binding proteins
CLN	ceroid lipofuscinosis neuronal
CTS	cathepsin
NCL	Neuronal ceroid lipofuscinosis
